# The association between Diabetes mellitus and Depression

**Published:** 2016

**Authors:** SV Bădescu, C Tătaru, L Kobylinska, EL Georgescu, DM Zahiu, AM Zăgrean, L Zăgrean

**Affiliations:** * Division of Physiology & Fundamental Neuroscience, “Carol Davila” University of Medicine and Pharmacy, Bucharest, Romania; ** Division of Ophthalmology, ”Carol Davila” University of Medicine and Pharmacy, Bucharest, Romania; Emergency Eye Hospital Bucharest, Romania

**Keywords:** diabetes mellitus, depression, comorbidity, epidemiology

## Abstract

Depression occurrence is two to three times higher in people with diabetes mellitus, the majority of the cases remaining under-diagnosed. The purpose of this review was to show the links between depression and diabetes, point out the importance of identifying depression in diabetic patients and identify the possible ways to address both diseases. Possible common pathophysiological mechanisms as stress and inflammation were explained, while emphasis was made on screening for depression in diabetic patients. An important aspect for the diabetic specialist would be the understanding of the common origins of diabetes and depression and the awareness of this quite common comorbidity, in order to improve the outcomes of both diseases.

**Abbreviations**:

DALYS = disability adjusted life years, DSM-5 = American Psychiatric Association Diagnostic and Statistical Manual of Mental Disorders, DM1 = Type 1 diabetes mellitus, DM2 = Type 2 diabetes mellitus, HPA-axis = hypothalamus – pituitary – adrenal axis, SNS = sympathetic nervous system, BDI = Beck Depression Inventory, CES-D = Centre for Epidemiologic Studies Depression Scale, HADS = Hospital Anxiety and Depression Scale, PHQ = Patient Health Questionnaire.

## Introduction

According to the International Diabetes Federation “diabetes is one of the largest global health emergencies of the 21st century” [**1**]. In 2015, the prevalence of diabetes worldwide was of one in 11 adults and the estimated prevalence of the impaired glucose toleration was of one in 15 adults. These numbers are expected to further increase, especially in the urban population, leading to more medical and economic challenges, added on top of the 12% global health expenditure currently spent on diabetes [**1**]. A recent study conducted in the Romanian population showed that diabetes is one of the major health care problems for our medical system, as its prevalence is of 11.6% and the prediabete’s one is of 16.5% [**2**]. 

Depression is a common and very serious medical disease with a lifetime prevalence ranging from approximately 11% in low-income countries to 15% in high-income countries [**3**]. The risk of having a mental health problem in life is of about 50% and this leads to a drop in employment, productivity and wages [**4**]. Depression and anxiety are the 4th cause, while diabetes is the 8th cause of disability adjusted life years (DALYS) in developed countries [**5**].

As it is defined by the American Psychiatric Association Diagnostic and Statistical Manual of Mental Disorders (DSM-5), diabetes is a mood disorder that reunites several symptoms that alter the functionality of an individual [**6**]. Depression disturbs emotions, cognition, and behaviors [**6**]. According to DSM–5, the diagnostic criteria for a major depressive disorder consist of a core symptom – either a diminished/ irritable mood or decreased interest/ pleasure (anhedonia) – or both, and at least four of the following symptoms: feelings of guilt or worthlessness, fatigue or loss of energy, concentration problems, suicidal thoughts or thoughts about death, weight loss or weight gain (5% change in weight), psychomotor retardation or activation (change in activity), hypersomnia or insomnia (change in sleep) lasting for at least 2 weeks [**6**]. Depression could be described as a first episode, a recurrent or chronic episode; could be mild, moderate or severe, with or without psychotic features [**6**]. 

There is evidence that the prevalence of depression is moderately increased in prediabetic patients and in undiagnosed diabetic patients, and markedly increased in the previously diagnosed diabetic patients compared to normal glucose metabolism individuals [**7**]. The prevalence rates of depression could be up to three-times higher in patients with type 1 diabetes and twice as high in people with type 2 diabetes compared with the general population worldwide [**8**]. Anxiety appears in 40% of the patients with type 1 or 2 diabetes [**9**]. The presence of depression and anxiety in diabetic patients worsens the prognosis of diabetes, increases the non-compliance to the medical treatment [**10**], decreases the quality of life [**11**] and increases mortality [**12**].

On the other hand, depression may increase the risk of developing type 2 diabetes with 60% [**13**,**14**].

It seems that there is a bidirectional association between diabetes and depression, a complex relation that might share biological mechanisms, whose understanding could provide a better treatment and improve the outcomes for these pathologies [**15**,**16**]. The purpose of this review was to show the connections between depression and diabetes, point out the importance of identifying depression in diabetic patients and the possible ways to address both diseases.

## Pathophysiological mechanisms

Due to the negative aspects regarding individual’s health and also health care systems, the co-morbidity of diabetes and depression have triggered many studies in the last decade. In 2015, two different reviews [**15**,**16**] indicated three possible directions for the association of diabetes and depression: both diseases might have a common etiology, diabetes increasing the prevalence or risk for future depression; depression increasing the prevalence or risk for future diabetes.

The median age of onset of depression, early to the middle 20s [**17**], and the different management therapy and age of onset for type 1 and 2 diabetes demand two separate approaches for the diseases’ co-morbidity. Type 1 diabetes mellitus (DM1) appears in childhood and early adulthood demanding daily insulin injections for life, while type 2 diabetes mellitus (DM2) appears later in life, in mid-adulthood, demanding diet and lifestyle modifications, oral medication or insulin injections [**16**].

Recent studies showed that there are not any common genetic factors to account for the positive association between depression and type 1 [**18**] or 2 [**18**,**19**] diabetes. 

However, different environmental factors (epigenetic factors) may activate common pathways that promote DM2 and depression in the end. One important factor is a low socioeconomic status that increases the odds for DM2 [**20**], but also appears to be a cause for depression [**21**]. The other common causes for DM2 and depression are poor sleep, lack of physical exercises and diet. Taking into consideration these factors, a key candidate for a common pathway could be the activation and disturbance of the stress system. Chronic stress activates the hypothalamus – pituitary – adrenal axis (HPA-axis) and the sympathetic nervous system (SNS), increasing the production of cortisol in the adrenal cortex and the production of adrenalin and noradrenalin in the adrenal medulla [**22**]. Chronic hypercortisolemia and prolonged SNS activation promote insulin resistance, visceral obesity and lead to metabolic syndrome and DM2 [**23**]. On the other hand, chronic stress has behavioral consequences: noradrenalin, cortisol and other hormones activate the fear system determining anxiety, anorexia or hyperphagia; the same mediators cause tachyphylaxis of the reward system, which produces depression and cravings for food, other substances or stress [**23**]. Excess cortisol disturbs neurogenesis in the hippocampus [**24**], a region involved in depression as well as in DM2 [**25**]. 

Moreover, chronic stress induces immune dysfunction directly or through the HPA axis or SNS, increasing the production of inflammatory cytokines. High amounts of inflammatory cytokines interact with the normal functioning of the pancreatic β-cells, induce insulin resistance, and thus, promote the appearance of DM2 [**26**,**27**]. Many new studies suggest that inflammatory responses are also involved in the pathophysiology of depression. Proinflammatory cytokines have been found to interact with many of the pathophysiological domains that characterize depression, including neurotransmitter metabolism, neuroendocrine function, synaptic plasticity, and behavior [**28**]. 50% of the patients treated with interferon Alfa develop depression and patients with depression had statistically higher blood levels of cytokines like tumor necrosis factor and interleukin 6 than those without depression [**28**].

These correlations suggested that stress (through the chronic impairment of HPA axis and SNS) and inflammation both promote depression and DM2, giving a feasible common link between them.

Patients with DM1 need a different and more complicated management of their disease compared with DM2: they need a frequent monitoring of their glycemia, adjusting insulin doses accordingly, diet and physical activity. The age of onset of DM1 is much earlier than for DM2; the close chronological relation between DM1 and onset of depression is striking, diagnosis of DM1 and its treatment burden occur in a period when the individual has an increased vulnerability to depression [**16**]. Children and adolescents with diabetes have a two to three times greater prevalence of depression than youth without diabetes [**29**]. A poor glycemic control in pediatric DM1 is related with both depression and lower socioeconomic status and the chances of depression in these patients increase as glycemic control worsens [**30**]. There are not so many studies on DM1 and depression, but one important review on the subject evidences a biological link: increased circulating cytokines associated with autoimmune diabetes, the lack of insulin affecting neurogenesis and neurotransmitter metabolism, the effects of chronic hyperglycaemia and those of iatrogenic hypoglycaemia and a hyperactivity in the HPA axis [**31**]. Similarly to DM2, it seems that DM1 and depression have common pathophysiological pathways, contrary to what it was traditionally thought, that the burden of diabetes increases the prevalence of depression [**31**].

## Diabetic risk in depressed patients

Several studies admitted that patients with depression have an increased risk of developing DM2 [**13**,**14**]. However, apart from the mechanisms explained earlier, other causes have been proposed. A recent study regarding the association between the antidepressant use and the glycemic control showed that in adults with diabetes, the use of multiple antidepressant subclasses increased significantly the levels of Hb A1C, suggesting that anti-depressive treatment may be a risk factor for suboptimal glycemic control [**32**]. Prior studies suggested that short-term anti-depressive treatment of nondiabetic depressed patients has a beneficial effect and improve insulin sensitivity together with improving depression, but on the long run, the effects might be opposite [**33**]. Noradrenergic antidepressants are an exception and may lead to impaired insulin sensitivity even in nondiabetic patients [**33**]. Selective serotonin reuptake inhibitor treatment may improve the glycemic control in depressed DM2 patients and is the only class of antidepressants with confirmed favourable effects on glycemic control on both short and long term use [**34**]. Continuous antidepressant use significantly associates with diabetes risk, making antidepressants rather than depression related to the incidence of DM2 [**35**]. It is important to understand the possible negative effects of antidepressant drugs on glycemic control and to try to minimize them.

When examining the effect of diabetes upon depressed patients, a 2015 study on 200936 depressed patients showed that comorbid DM could increase the risk of complications of depression, such as suicide and hospitalization [**36**].

## Depression risk in diabetic patients

A recent epidemiological study of 90686 participants found that depression was more prevalent in people with diabetes, regardless of the fact that they had diagnosed or undiagnosed diabetes. The same study showed that anxiety was more prevalent only in participants who were aware of their diabetes [**37**]. One possible explanation might be that the psychological burden of being ill may play an important role on triggering anxiety and depression. However, the fact that in patients with previously undiagnosed diabetes, depression had a higher prevalence and could be due to an unfavourable lifestyle, such as physical inactivity, unhealthy diet or a stressful lifestyle.

Severe hypoglycaemia in patients with DM2 and without anti-depressive treatment was positively associated with the severity of depressive symptoms, independent of glycemic control, insulin therapy, lifestyle factors and diabetic complications [**38**]. A meta-analysis estimating the association between depression and neuropathy in patients with DM2 could not clarify if the relationship is bidirectional or not [**39**].

Hypothetically, depression could be increased by anti-diabetic treatment [**15**]. A strong association between depression in patients in their forties with orally treated diabetes was found, compared to patients in their seventies [**40**]. On the contrary, insulin therapy in elderly people with DM2 led to the improvement of depressive symptoms and did not affect the health-related quality of life of these patients [**41**]. 

Diabetes produces structural changes in the brain: cerebral atrophy and lacunar infarcts, blood flow changes of both hypo- and hyperperfusion [**42**]. Reductions in brain volumes restricted to the hippocampus were found in patients with diabetes, while an inverse relationship between glycemic control and hippocampal volume was present. HbA1C was described as the only significant predictor of hippocampal volume [**43**]. Similarly, depression is associated with neurodegenerative processes, especially at the level of the prefrontal cortex and hippocampus [**44**]. The enhancement of indoleamine 2, 3-dioxygenase enzyme activity with the kynurenine pathway activation and increased synthesis of interferon-stimulated gene products involved in the apoptotic process (Tumor necrosis factor-α-related apoptosis-inducing ligand, caspase-4, caspase-8, and death activating protein kinases) seems to be the principal mechanisms involved in the neurodegeneration-depression process induced by chronic inflammation [**45**-**47**]. 

## Consequences of diabetes and depression in clinical practice

Depression has a synergistic effect in patients with DM1 and DM2, increasing the risk for complications of both micro- and macro-vascular nature, increased hyperglycemia, predicting greater mortality. In older adults, the comorbidity also predicts an earlier incidence of complications [**48**]. Both diabetes and depression reduce the quality of life for an individual, but together they have a more negative impact [**49**]. Due to the negative effects on health, the rise in complications, both diseases should be recognized in an individual and treated simultaneously, in order to reduce depression and better control the diabetes. However, depression remains under-diagnosed and untreated in diabetic patients [**50**]. Increased awareness for depression in diabetes might improve the outcomes and a first step would be a simple method for screening depression to be used on regular diabetic follow-ups. 

Screening questionnaires for depression might give an overestimation of depression [**51**], but they are a simple and quick method. Therefore, positive screening should be validated by an interview with a specialist in mental care. Among the many short questionnaires that have been used to detect depression, the Beck Depression Inventory (BDI) and the Centre for Epidemiologic Studies Depression Scale were the most popular ones (CES-D), followed by the Hospital Anxiety and Depression Scale (HADS) and different versions of the Patient Health Questionnaire (PHQ) [**52**]. The PHQ–9 is the most used and validated screening test for depression in people with diabetes with a high sensitivity and specificity [**53**]. As a screening instrument for depression in diabetes patients, a study for the validation of the PHQ-9 suggested that increasing the cut-off for major depression at ≥ 12 points (instead of 10 points) in diabetic patients may improve the discrimination between diabetes related symptoms and depressive symptoms [**54**].

When depression is diagnosed in a diabetic patient, the common sense would be to treat both diseases at the same time. Petrak et al. recommended treating depression as a priority, as the response to medication is usually seen within 2-4 weeks for antidepressants, while the improvement in the glycemic control and levels of HbA1C needs several months to settle [**55**]. Moreover, Petrak et al. suggested that patients having a better mood might follow their diabetic treatment better [**55**]. They also proposed a model for treating depression and diabetes, stepped according to the degree of depression [**55**].

A new approach would be to identify the common triggers for diabetes and depression, and try to address them, but further studies should be done in this direction – controlling or preventing stress and inflammatory responses. Lifestyle changes such as increased physical activity or exercise, dietary modification, adequate relaxation/ sleep and social interaction, use of mindfulness-based meditation techniques, and the reduction of recreational substances such as nicotine, drugs, and alcohol already proved their benefits in the improvement of depression as well as diabetes [**56**]. 

## Conclusions 

For a healthy society, it is important to prevent, identify, and treat the health problems. However, the World Health Organization warns us that there is “a substantial gap between the burden caused by mental disorders and the resources available to prevent and treat them. It is estimated that four out of five people with serious mental disorders living in low and middle income countries do not receive the mental health services that they need” [**57**]. In diabetic patients, depression remains underdiagnosed and an important aspect for the diabetic specialist would be the awareness of this quite common co-morbidity. A multidisciplinary approach of the diabetic patient would help improve the outcomes of disease, decrease the number of DALYs and even mortality.

**Sources of funding**

The study was supported by “Carol Davila” University of Medicine and Pharmacy through the “Young Researchers” Grant no. 28482/ 2012.

**Disclosures**

None
